# Considering intermittent fasting among Saudis: insights into practices

**DOI:** 10.1186/s12889-022-12908-4

**Published:** 2022-03-26

**Authors:** Aroub Alnasser, Mashael Almutairi

**Affiliations:** Food Science and Nutrition Department, College of Food and Agriculture Sciences, King Saud University, P.O. Box 22452, Riyadh, 11495 Saudi Arabia

**Keywords:** Intermittent fasting, Saudis, Ramadan, Cross-sectional, Saudi Arabia, Fasting

## Abstract

**Background:**

There is a dearth of studies on intermittent fasting in Saudi Arabia outside of Ramadan. The aim of this research was to study and describe the practice of intermittent fasting outside of Ramadan among Saudi people.

**Methods:**

A web-based survey that focused on intermittent fasting practices—specifically the use of intermittent fasting applications, goal setting, and the effects of fasting on an individual’s state of health—was administered, collected, and analyzed.

**Results:**

The study revealed that 58% (298/514) of the respondents practiced intermittent fasting for a duration of less than 3 months. The most-practiced pattern of intermittent fasting was a 16/8 fasting pattern (43.8%, 225/514). About 88.3% (454/514) of those who followed intermittent fasting drank fluids while fasting. Additionally, the amount of weight loss after intermittent fasting was less than 2.2 kg for 35% (180/514) of the participants. The primary goal of intermittent fasting for 44.9% (231/514) of the respondents was to lose weight. The majority of the participants (84.6%, 435/514) did not use any fasting applications.

**Conclusion:**

The results of the current research on intermittent fasting outside of Ramadan are preliminary and inconclusive. The findings of the present study advance the idea that for some Saudis, the practice of intermittent fasting does not necessarily begin and end with Ramadan; this finding may present a strategic opportunity for Saudi health professionals who are focused on the obesity epidemic and other public health issues in Saudi Arabia. This study sought to help start a discussion on this topic and fill the knowledge gap.

**Supplementary Information:**

The online version contains supplementary material available at 10.1186/s12889-022-12908-4.

## Background

Fasting practices have been part of human life for religious, cultural, or health reasons for thousands of years. For Muslims, the culturally intrinsic practice of fasting has been around for centuries. The Muslim community worldwide, including Muslims in Saudi Arabia, fasts for an entire lunar month during Ramadan.

Islamic fasting studies based in Muslim countries have particularly focused on intermittent fasting during Ramadan [1–6]. Although the research [7–12] has included an analysis of practices and outcomes, these studies were also limited to a focus on intermittent fasting *during* Ramadan.

Furthermore, the majority of the research on intermittent fasting based in Muslim countries has been limited to the study of the physiological effects of fasting during Ramadan, for example, the effect of fasting during Ramadan on diabetes treatment [10, 13, 14], cardiovascular disease [1, 15, 16], body weight [17–19], athletic performance [20–22] and sleep [23].

A review of the current international literature reveals very limited descriptive research focused on intermittent fasting practices. As Cuccolo notes [24], there is a wealth of literature focused very specifically on whether and how intermittent fasting produces clinical changes in different contexts and with different biomarkers. For instance, in terms of lipid profiles, intermittent fasting was shown to be effective in improving total cholesterol, low-density lipoprotein cholesterol, and triacylglycerol concentrations [25, 26]. Intermittent fasting was associated with greater weight loss in patients with diabetes compared with a regular diet [27]. In terms of body composition, intermittent fasting was positively correlated with short-term weight loss [28–30]. Despite the abundance of specifically targeted studies concentrated on the apparent and ranging physiological advantages of intermittent fasting, there is limited research that is more broadly directed at intermittent fasting practices, and the implementation, motivation, and perceptions of these practices. Moreover, because these and other studies have confirmed the multiple and varying benefits of intermittent fasting for human health, this body of research is pertinent to the current study and relevant for the continuation and expansion of a dialog on intermittent fasting practices.

In terms of motivation and perceptions, the study by Potter and colleagues [31] compared individuals who implemented intermittent fasting in the past and individuals who were currently practicing intermittent fasting, revealing that a person’s beliefs about a traditional three-meals-a-day lifestyle may play a key role in adherence to dietary practices.

Given the limited literature, little is still known about how people in general and the Saudi community, in particular, are practicing intermittent fasting. Therefore, to fill the research gap and augment the limited body of descriptive research on intermittent fasting, the aim of this research was to study and describe the practice of intermittent fasting outside of Ramadan among Saudi people.

## Methods

### Study design andsample

This cross-sectional web-based survey was conducted with Saudi adults who used social networks. The questionnaire link was publicized through social media (Twitter, WhatsApp, and Telegram), and responses were collected over 1 month. When participants accessed the web-based survey, they were greeted with the name and purpose of the study, as well as a statement about the anonymity and confidentiality of the information collected. Next, the screening criteria (18 years old or older, practicing intermittent fasting outside of Ramadan, etc.) were presented. If a participant met the criteria, s/he was directed to a statement of informed consent that asked them to check agree/disagree. Once a participant agreed to provide informed consent, s/he was able to complete the survey.

All information—from the study name to the final question—was presented textually. The participants reported no issues with survey access, understanding the language within the study, or the informed consent statement.

The inclusion and exclusion criteria of the study and the responses given by the participants are shown in Table 1.

**Table 1:** The inclusion and exclusion criteria of the study and the responses given by the participants

Inclusion/Exclusion	Category	Criteria
Inclusion	Survey period	11 March 2021–8 April 2021
	Nationality	Saudi
	Sex	Male or female
	Age	At least 18 years old
	Region of research	Saudi Arabia, but Saudis living abroad could also participate
	Methodology	Web-based questionnaire
	Topic of research	Intermittent fasting practices among Saudis
Exclusion	Age	Younger than 18 years
	Responses	Responses that were illogical or incomplete
	Intermittent fasting	Participants that did not practice intermittent fasting

A sample size of 385 was generated via the Raosoft sample size calculator [32] with a 95% confidence level, 5% margin error and response rate of 50%, which was comparable to another Saudi-based study [33], to represent Saudi Arabia’s current population of 12.6 million adults aged ≥ 18 years old [34].

### Questionnaire development

Given the scarcity of descriptive studies on intermittent fasting practices, as noted by Cuccolo and colleagues [24], a questionnaire was developed with the aim of addressing this gap and to better understand intermittent fasting practices. The questionnaire was created after reviewing the existing literature. An examination of multiple sources yielded a pool of questions deemed relevant to the study goals [31, 35].

Regarding content validity, the questionnaire was sent to five health and academic professionals who had collectively published numerous studies that included survey-based research. Based on their recommendations, the survey was rewritten for clarity, relevance, and consistency. For the evaluation of face validity, before conducting the study, the questionnaire was tested with a sample of Saudi adults (*n* = 10) and was revised based on their feedback. Finally, during the piloting process of the questionnaire, Cronbach’s alpha was used to determine internal consistency reliability. A good value of 0.80 was obtained. The final web-based questionnaire was published on Google Forms with the idea that answers to the questions and respondent feedback could be given in real time.

The questionnaire consisted of 22 close-ended questions (Additional file 1) that encompassed the following determinants and was developed in compliance with the Checklist for Reporting Results of Internet E-Surveys (CHERRIES) guidelines [10, 36] and the Strengthening the Reporting of Observational Studies in Epidemiology (STROBE) guidelines [37]:

1. Demographic characteristics (age, sex, residency, education, employment status, weight, height and medical diagnoses)
2. Intermittent fasting practices (patterns of intermittent fasting, type of food)
3. The effect of fasting on a person’s state of health (post-fasting portion sizes, weight loss, hunger, health status, physical symptoms)
4. Fasting goals (cause and duration of the practice, plans, sources of knowledge about intermittent fasting, resources on intermittent fasting)
5. Use of technology in fasting (name of the app if applicable)

### Data analysis

Data were analyzed using the Statistical Package of Social Sciences (SPSS) system (Version 22.0). Descriptive statistics (frequency, percentages, and cross-tabulation), the chi-square test, and linear regression were applied. The *P* value significance was set at *P* < 0.05 and 95% confidence intervals (CIs) were reported.

## Results

### Socioeconomic characteristics and health status

The survey was completed by 575 respondents according to the inclusion and exclusion criteria (shown in Table 1). Sixty-one respondents reported information (age, height, etc.) that excluded them from the study. Even after identifying as Saudi, over 18 years of age, and practicing intermittent fasting outside of Ramadan, participants were asked in two later survey checkpoint questions if they 1) practiced intermittent fasting outside of Ramadan and 2) to report their specific age and height for the calculation of body mass index (BMI); 47 respondents reported that they did not practice intermittent fasting outside of Ramadan, which excluded them from the study, and ten respondents entered values for height or age that excluded them from the study. Ultimately, 514 respondents were selected for analysis, of which 11.9% (61/514) were male and 88.1% (453/514) were female. The majority of the respondents were aged between 18 and 31 years (56.2%, 289/514) and were residents of the central region of Saudi Arabia, including Riyadh (37.7%, 194/514). It was interesting to observe that 67.3% (346/514) of the respondents considered themselves to be in good or excellent health, and nearly the same percentage of respondents either reported themselves as being overweight or suffering from moderate or severe obesity, or were calculated as such based on their reported height and weight. These results are in agreement with findings from the Saudi National Survey, which showed that risk factors such as obesity were not associated with Saudis’ self-rated health [38].

The most prevalent medical conditions that the respondents reported were obesity (7.6%, 39/514), high blood cholesterol and triglyceride levels (5.3%, 27/514), and hypothyroidism (2.3%, 12/514). A total of 30.5% (157/154) of the respondents had a BMI in the normal range, and 67.3% (346/514) had a BMI in the overweight or moderate or severe obesity range (as shown in Table 2).

**Table 2:** Sociodemographic characteristics of the study subjects by sex

Variables	Sex		Total	***P*** Value
	Female	Male		
**Age, years** (mean ± SD)	31.43 ± 8.89	35.23 ± 10.59	31.88 ± 9.18	0.002*
**Age group** (years)				
18–31	264 (51.4%)	25 (4.9%)	289 (56.2%)	0.013*
32–45	154 (30.0%)	26 (5.1%)	180 (35.0%)	
46 and older	35 (6.8%)	10 (1.9%)	45 (8.8%)	
**Region of the Country**				
Central	173 (33.7%)	21 (4.1%)	194 (37.7%)	0.134*
Southern	47 (9.1%)	7 (1.4%)	54 (10.5%)	
Eastern	132 (25.7%)	11 (2.1%)	143 (27.8%)	
Northern	21 (4.1%)	6 (1.2%)	27 (5.3%)	
Western	7 (13.8%)	13 (2.5%)	84 (16.3%)	
Living abroad	9 (1.8%)	3 (0.6%)	12 (2.3%)	
**Education level**				
Up to high school	85 (16.5%)	15 (2.9%)	100 (19.4%)	< 0.001**
Undergraduate	322 (62.3%)	40 (7.7%)	362 (70.4%)	
Graduate and postgraduate	46 (8.9)	6 (1.2%)	52 (10.1%)	
**Employment**				
Student	99 (19.3%)	9 (1.8%)	108 (21.0%)	< 0.001**
Employee	141 (27.4%)	42 (8.2%)	183 (35.6%)	
Not employed	192 (37.4%)	4 (0.8%)	196 (38.1%)	
Retired	21 (4.1%)	6 (1.2%)	27 (5.3%)	
**BMI**				
Underweight	10 (1.9%)	1 (0.2%)	11 (2.1%)	0.302*
Normal weight	137 (26.7%)	20 (3.9%)	157 (+ 30.5%)	
Overweight	184 (35.8%)	19 (3.7%)	203 (39.5%)	
Obesity	122 (23.7)	14 (4.1%)	143 (27.8%)	
**Have you ever been diagnosed with any of the following conditions by a health professional?**				
None	315 (61.3%)	31 (6.0%)	346 (67.3%)	0.01*
Yes	138 (26.8%)	30 (5.9%)	168 (32.7%)	
**Total Count (%)**	453 (88.1%)	61 (11.9%)	**514 (100%)**	

*P* < 0.05: Significant, * statistically significant, ** highly statistically significant

### Intermittent fasting practices

The survey focused on intermittent fasting practices with a particular focus on four factors: months/years of fasting, the fasting practice pattern, hydration during fasting (if any), and the diet plan. A duration of less than 1 month (32.5%) and a 16 h fasting pattern (43.8%, 225/514) was the most practiced approach among Saudis. Intermittent fasting for one to 3 months (25.5%) was the most prevalent duration, and a 12-h (26.3%, 135/514) fasting pattern was the second most prevalent pattern.

**Fig. 1:**
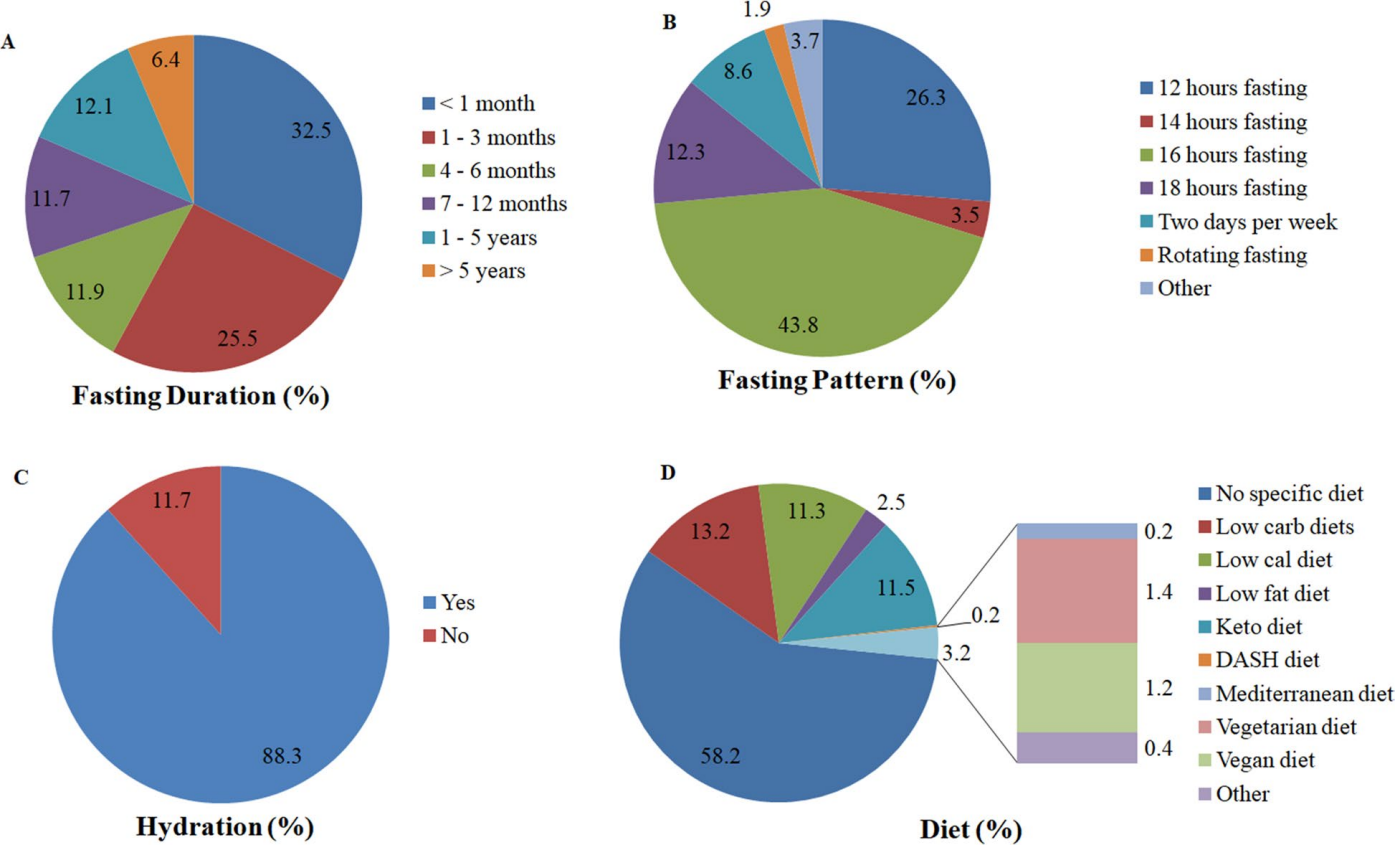
Fasting practices among Saudi adults. **A** Fasting duration, **B** Fasting pattern, **C** Hydration status and **D** Diet. Data are represented as an overall percentage

In terms of hydration during fasting, 88.3% (454/514) of the respondents kept themselves hydrated by drinking fluids during intermittent fasting. On the other hand, 11.7% (60/514) of the respondents did not drink any fluids for religious reasons (63.3%, 38/60), because they forgot to hydrate (11.6%, 7/60) and/or (25%, 15/60) or for other reasons such as sleep and laziness. The results related to intermittent fasting patterns, practices, and diets are shown in Fig. 1.

It was noteworthy that among the respondents, the majority of Saudi adults did not follow any specific diet plan (58.2%, 299/514). Only a small number of respondents followed a specialized diet plan, such as a low-carb diet (13.2%, 68/514), a keto diet (11.5%, 59/514), and a low calorie diet (11.3%, 58/514).

### The effect of fasting on state of health

To evaluate the full impact of intermittent fasting, weight loss was studied. The physical impact of intermittent fasting on eating habits, health status, and the changes felt by the participants were also studied. Most participants (62.5%, 321/514) reported consuming less food than usual. The eating habits of 34% (175/514) of the respondents were not changed. However, 18 (3.5%) participants responded that they ate more food than usual after intermittent fasting, and 60.7% (312/514) of the participants lost less than 5 kg after intermittent fasting (as shown in Table 3).

**Table 3:** Impact of fasting on the participants’ health

Variables	Total
**After intermittent fasting, is the amount of food you eat:**	
Less than usual	321 (62.5%)
Unchanged	175 (34.0%)
More than usual	18 (3.5%)
**After intermittent fasting, did your weight change, and by how much?**	
Less than 2.2 kg	180 (35.0%)
2.2–4.9 kg	132 (25.7%)
5–9.9 kg	52 (10.1%)
10–14.9 kg	15 (2.9%)
15–19.9 kg	4 (0.8%)
More than 2.0 kg	16 (3.1%)
I did not lose weight	109 (21.2%)
I gained weight	6 (1.2%)
**After practicing intermittent fasting, my level of hunger has become**	
Less	383 (74.5%)
More	28 (5.4%)
Unchanged	103 (20.0%)
**After intermittent fasting, my health status is:**	
Better	315 (61.3%)
Worse	17 (3.3%)
Unchanged	179 (34.8%)
Other - please specify	3 (0.6%)
**Have you experienced any of these physical symptoms during fasting?**	
Hunger	38 (7.4%)
Lethargy	47 (9.1%)
Headaches	25 (4.9%)
Feeling dizzy	16 (3.1%)
Constipation	14 (2.7%)
Sensation of being cold	11 (2.1%)
I had no symptoms.	188 (36.6%)
I had more than one symptom	175 (34.0%)
**Total Count (%)**	**514 (100%)**

The majority of the participants (61.3%, 315/514) felt that their health was improved after practicing intermittent fasting, and 34.8% (179/514) did not feel they experienced any changes.

In terms of reported physical symptoms during intermittent fasting, 36.6% (188/514) of the participants did not experience any physical effects during intermittent fasting, and a small number (7.4%, 38/514) experienced hunger while practicing intermittent fasting.

### Fasting goals and the use of technology

The primary goal of practicing intermittent fasting was to lose weight among 44.9% (231/514) of the respondents who reported that they wanted to lose some weight (as shown in Table 4). However, the rest of the respondents practiced intermittent fasting for multiple reasons, such as the optimization of health preservation and religious reasons. In addition, approximately 36.8% (189/514) of the participants were planning to continue practicing fasting until they reached their goals, and 21.4% (110/514) planned to continue to practice intermittent fasting for the rest of their lives. It is possible that the simplicity of the intermittent fasting regimen—as well as the complex relationship among intermittent fasting, religious practices, health, weight goals, and lifestyle—may explain why more than one-fifth of the participants planned to continue intermittent fasting throughout their lives.

**Table 4:** Knowledge about fasting goals and the use of technology among the study population

Variables		Total
**Why do you practice intermittent fasting?**		
To lose weight		231 (44.9%)
To be healthy		56 (10.9%)
Religious reasons		36 (7.0%)
Multiple reasons		172 (33.5%)
Other		19 (3.7%)
**How long do you plan to continue to practice fasting?**		
< 1 year		52 (10.1%)
> 1 year		26 (5.1%)
Until I reach my goals		189 (36.8%)
For the rest of my life		110 (21.4%)
I do not know		137 (26.7%)
**How did you learn about this practice of fasting?**		
From a health program on TV		24 (4.7%)
Through social networking sites		320 (62.3%)
Discussed with health professionals		33 (6.4%)
Through friends and family		105 (20.4%)
Other		21 (4.1%)
**Which app do you use for fasting?**		
Zero		28 (5.4%)
Body Fast		5 (1.0%)
In Fasting		2 (0.4%)
LIFE Fasting Tracker		10 (1.9%)
Fastient		2 (0.4%)
Fast Habit		4 (0.8%)
I do not use any app for fasting		435 (84.6%)
Other		28 (5.4%)
**If you responded no to the last question, why don’t you use an intermittent fasting phone app?**		
No applications in Arabic are available		17 (3.3%)
I did not know there were applications for intermittent fasting		192 (37.4%)
I do not need apps to fast		226 (44.0%)
**Total Count (%)**		**514 (100%)**

The majority (62.3%, 320/514) of the respondents learned about the practice of fasting through social networking sites, while a minority (6.4%, 33/514) learned through discussion with health professionals. Almost 84.6% (435/514) of the participants did not use any smartphone application for fasting, while 37.4% (192/514) were not even aware of applications that help manage intermittent fasting. Most participants who used intermittent fasting management applications reported using the Zero app (35.4%, 28/79).

### Body mass index (BMI)

BMI is a metric used by clinicians and public health organizations in predicting and determining overweight and obesity (weight status category). In addition to self-reported weight change (or stasis), BMI was used for determining any post-intermittent fasting weight changes. Linear regression was used to determine the interaction between the fasting status and pattern and hydration status with BMI among the study population (F = 8.199, *p* < 0.001; adjusted R-Square = 0.040; as shown in Table 5). Approximately 4.0% of the variance in the BMI scores could be explained by the fasting status and pattern and hydration status of the participants. The findings show that the overall BMI status of the respondents was significantly (*P* < 0.05) affected by practicing intermittent fasting for a long period of time and by hydration status (*P* < 0.001 and 0.005, respectively). Respondents who were not new to intermittent fasting had a significantly lower BMI (OR = 0.55) than those who had not practiced intermittent fasting. In addition, respondents who hydrated during fasting had a significantly lower BMI than those who did not hydrate during fasting (OR = 2.3).

**Table 5:** The interaction between fasting status and pattern with BMI among the study population

a	Predictors	Beta	95% confidence intervals beta coefficient	***P*** value	Adjusted R	F	***P*** Value
**BMI**	For how long have you practiced intermittent fasting?	−0.55	−0.870 to −0.241	< 0.001	0.040	8.199	< 0.001**
	What is your intermittent fasting pattern?	0.199	−0.142 to 0.541	0.289			
	Are you hydrating during fasting?	−2.39	−3.996 to − 0.792	0.005			

a. Dependent variable (BMI), ** highly statistically significant

### The duration of intermittent fasting practices

Moreover, linear regression was used to determine the effect of sociodemographic characteristics on the duration of intermittent fasting practices among the study population (F = 4.901, *p* < 0.001, adjusted R-squared = 0.037). Approximately 3.7% of the variance in intermittent fasting practices could be explained by the sociodemographic characteristics of the participants (Table 6). The findings show that the overall duration of the respondents’ intermittent fasting practices was significantly (*P* < 0.05) affected by the sex and age of the participants (*P* value = 0.002 and 0.008, respectively). However, women practiced longer periods of intermittent fasting than men (OR = 0.143). Furthermore, older respondents practiced longer periods of intermittent fasting than younger respondents (OR = 0.123) (Table 6).

**Table 6:** The effect of sociodemographic characteristics on the duration of intermittent fasting practices among the study population

a	Predictors	Beta	95% confidence intervals beta coefficient	***P*** value	Adjusted R	F	***P*** Value
The duration of intermittent fasting practices	Sex	0.143	0.274 to 1.158	0.002*	0.037	4.901	< 0.001**
	Age	0.123	0.006 to 0.038	0.008*			
	Region	−0.071	−0.165 to 0.016	0.108			
	Education level	0.030	−0.097 to 0.198	0.502			
	Employment status	0.034	−0.080 to 0.172	0.476			

a. Dependent variable (the duration of intermittent fasting), * statistically significant, ** highly statistically significant

## Discussion

The study sought to explore intermittent fasting patterns among a sample of Saudis to better understand the practice of intermittent fasting. Intermittent fasting has shown an increase in popularity as a method for weight loss and health optimization, and previous research has found promising results for its utility [39]. The current study collected data on intermittent fasting duration and habits, as well as health perceptions, and aggregated data exploring intermittent fasting trends and impacts. Unsurprisingly, although this study was not focused on sex, it should be noted that the majority of the participants were female; it is well known that males are less likely to participate in surveys [40, 41].

The results found in the present study align with the previous literature in a myriad of ways. Most participants (43.8%) reported that they followed a 16/8 fasting pattern, which is supported by a recent systematic review [30]. Welton and colleagues reported that the ideal period of a fast for positive weight change appears to be 16 h [24, 30]; our results are in accordance with these findings and indicate that the optimum intermittent fasting period is already inherent to Saudi intermittent fasting practices. Furthermore, consistent with the findings of previous research that noted that long-term intermittent fasting and hydration with noncaloric beverages contributed to a reduction in BMI and stomach circumference [39], we observed that the overall BMI status of the respondents was significantly affected (*P* < 0.05) by the hydration status and duration of intermittent fasting. Finally, Haines [42] found, as the current study concluded, that at least 50% of participants planned to continue the practice of intermittent fasting for some duration beyond the time of the survey.

In terms of studies focused on intermittent fasting in Arabic-speaking countries, in another study on voluntary fasting in Saudi Arabia [43], survey participants identified that the most common reasons for fasting were weight loss (47.59%; 44.9% in our study). However, another study on intermittent fasting in Saudi Arabia [35] also found that intermittent fasting was “useful and effective for weight loss.” The same study also identified a predisposition toward using intermittent fasting as a culturally familiar weight-loss tool in the Middle East and noted that intermittent fasting “was prevalent among Arabs because of their habituation toward it.” In the present study, more than half of the participants reported that they lost less than 5 kg after intermittent fasting. It is well known that a weight loss of 5% is defined as clinically meaningful weight loss that lowers the risk of many diseases [44] and can be a measure of lifestyle weight-loss program efficacy [45].

Sutton and colleagues [46] observed no significant change in self-reported hunger during a two- or four-week period of intermittent fasting. In another study [47], researchers noted a significant increase in participant hunger levels. However, our study found that after practicing intermittent fasting, the majority (75%) of the participants reported decreased hunger levels. A small number of our participants also reported other physical symptoms in response to intermittent fasting, such as headaches, dizziness, a sensation of being cold, and constipation after intermittent fasting.

Further clinical studies are warranted to assess the risks associated with long-term intermittent fasting, and factors such as protein malnutrition, vitamins, and mineral malnutrition should also be considered [48]. It has been reported that long-term intermittent fasting could adversely affect health, and due to insufficient energy intake, it may cause potential side effects such as dizziness, nausea, headaches, weakness, and excessive hunger [48]. Therefore, studies on the short- and long-term physical symptoms related to intermittent fasting have shown mixed results and justify more research and discussion.

Although most participants learned about the practice of fasting through social networking sites, surprisingly, smartphone applications did not play a significant role in intermittent fasting management for Saudis. Given that Saudi Arabia has the most social network users in the Middle East and is ranked second worldwide in terms of the percentage of social media users [49], clinicians should consider using social networking as a public awareness and community health information tool. A culturally adapted and religiously sensitive fasting application that is available in Arabic might attract more users from Arabic countries, particularly Saudi Arabia [50].

Study analyses have revealed that there is a relationship between hydration status and BMI. Our finding was in agreement with a previous national survey of 9528 adults [51] (Chang et al., 2016) that also found a strong association between poor hydration levels and a higher BMI. Another recent study among 8699 adults [52] from the National Health and Nutrition Examination Survey discovered a greater probability of hypohydration in participants with a BMI ≥30 kg/m2.

Our study revealed that the overall duration of intermittent fasting practices was significantly impacted by the sex and age of the participants. The results showed that women practiced longer periods of intermittent fasting than men. Additionally, the analyses pointed out that older respondents practiced longer periods of intermittent fasting than younger participants. In another study [53], researchers found a positive correlation between intermittent fasting and body composition in women aged 60 and older. Gupta & Singh [54], curiously, found that more men than women practiced intermittent fasting. Interestingly, contrary to our findings, they also observed that young adults practiced intermittent fasting more than middle-aged or older adults. Future research into sex, age, and intermittent fasting practices might help provide valuable insights into the sociodemographics of intermittent fasting practices and motivations.

The limitations of our study include the following. First, the use of purposive sampling was one of the study’s main limitations. Although this is a cost- and time-effective sampling strategy, its main limitation is that the results are not representative of the population and are not generalizable [55, 56]. Second, information bias, or reporting bias more specifically, may have negatively affected the accuracy of our survey, which was reliant on self-reported data. Self-reported data, such as weight loss and BMI data, might be biased and misreported. It is well documented [57] that self-reported body weight, for instance, is often underestimated. Additionally, because we could not independently verify participants’ self-reported values, self-reported bias must be considered. Third, 61 participants reported information that excluded them from the study, so this also limited the scope of the research and further limited any potential generalizability of the study. Fourth, the design of the Google Forms questionnaire did allow for the determination of a response rate. Finally, because of time and financial considerations and COVID-19 restrictions, the study relied on single-mode online data collection. As such, we could not ensure that a wider pool of potential participants was reached through a variety of survey opportunities and data collection modes. Additionally, online data collection may be a barrier for individuals with physical or internet accessibility issues, those who are not on social media or infrequent users of social media. Therefore, selection bias occurred in this study and is one of the study’s limitations. To overcome the limitations encountered in the present study and to gain a more in-depth understanding of intermittent fasting practices, future research should consider qualitative approaches, such as participant focus groups and interviews, as well as mixed-mode or multimodal data collection methods, such as mail, phone, and online survey access.

## Conclusion

This research sought to assess and better understand the Saudi practices of intermittent fasting outside of Ramadan, and hoped to continue the conversation on intermittent fasting practices. We discovered that most participants practiced intermittent fasting for a duration of less than 3 months. It was observed that 16-h intermittent fasting was the most common intermittent fasting practice among Saudi individuals. Additionally, weight loss was found to be the main purpose for practicing intermittent fasting. In fact, the data showed that intermittent fasting significantly reduced weight and BMI among participants who were practicing intermittent fasting for a long period of time, with or without hydration. Furthermore, our findings indicated that the majority of the participants were satisfied with the outcomes of their fasting practices and noted a general improvement in their health. Finally, smartphone applications did not play any notable role in intermittent fasting practices among Saudi people.

## Supplementary Information

**Additional file 1.**

## Data Availability

The data that supported the findings of this study are available from the corresponding author on request.

## Abbreviations

BMI: Body Mass Index
