# Supplementary material for: Considering intermittent fasting among Saudis: insights into practices
Source: BMC Public Health. 2022 Mar 26;22:592. doi: 10.1186/s12889-022-12908-4 (PMC8959076; doi:10.1186/s12889-022-12908-4)
Supplement: Supplementary file 1 — Additional file 1. [file 12889_2022_12908_MOESM1_ESM.docx]

**Title: “Considering intermittent fasting among Saudi: insights into practices”**

Do you follow intermittent fasting? (no food consumption)

If the answer is "yes," and you are Saudi, 18 years of age or older, and have practiced intermittent fasting currently or previously, we are inviting you to take a King Saud University Institutional Review Board approved research survey (reference No: KSU-HE-21-162) on intermittent fasting. The purpose of the survey is to receive feedback regarding the practice of intermittent fasting outside of Ramadan. Survey responses will help us better understand the practice of intermittent fasting in the Kingdom of Saudi Arabia.

This survey will take about three minutes, and participation is entirely voluntary. We do not collect any personal information, i.e., your name, email, or IP address. All survey data is secure and will be used exclusively for research purposes.

Please choose one of the following:

- Agree
- Disagree

**First, Demographic data**

1. **Gender**

- Female
- Male

1. **Age** - - - - - - - (years)
2. **Region of Country**

- Central
- Southern
- Eastern
- Northern
- Western
- Living abroad

1. **Education**

- Primary school degree
- Middle school degree
- High school degree
- Diploma
- Bachelor’s degree
- Graduate degree (Masters, PhD, MD, etc.)

1. **Employment**

- Student
- Employee
- Not employed
- Retired

1. **Weight** ………...in kg
2. **Height** …………. in cm
3. **Have you ever been diagnosed with any of the following conditions by a health professional?**

- None
- High fat or cholesterol
- High blood pressure
- Depression
- Diabetes
- Heart disease
- Obesity
- Other chronic diseases

**Second, Intermittent fasting practices**

1. **Do you or have you ever practiced fasting outside of Ramadan?**

- Yes
- No

1. **For how long have you practiced intermittent fasting?**

- < a month
- 1 - 3 months
- 4 - 6 months
- 7 - 12 months
- 1 - 5 years
- > 5 years

1. **What's your pattern of intermittent fasting?**

- 12 hours fasting, 12 hours eating
- 16 hours fasting: 8 Hours of Eating
- 18 hours fasting: 6 Hours of Eating
- Fasting two days a week
- Rotating fasting (day fasting and day eating)
- Other (please specify)

1. **Are you hydrating during fasting?**

- Yes
- No )why)?................

1. **What kind of food do you eat when you break your fast?**

- No specific diet.
- A Low carb diet.
- A Low-cal diet.
- A Low-fat diet.
- A Keto diet.
- A DASH diets
- A Mediterranean diet.
- A Vegetarian diet.
- A Vegan diet.

**Third, the effect of fasting on the individual’s state of health**

1. **After intermittent fasting, is the amount of food you eat...**

- Less than usual
- Same amount
- More than usual

1. **After intermittent fasting, how much weight did you lose?**

- Less than 2.2 kg
- 2.2-4.9 kg
- 5 - 9.9 kg
- 10-14.9 kg
- 15- 19.9 kg
- More than 2,000 kg
- I didn't lose weight.
- I gained weight.

1. **After practicing intermittent fasting, the level of hunger has become?**

- Less
- More
- It hasn't changed

1. **After intermittent fasting my health status?**

- Better
- It's deteriorated
- Hasn't changed
- Other (please specify)

1. **Have you experienced any of these physical symptoms during fasting like (You can choose more than 1)**

- Hunger
- Energy shortages and inactivity
- Headaches
- Feeling dizzy.
- Catch
- The Cold Sensation
- No, I had nothing.
- Other (please specify)

**Fourth: Fasting goal; cause and duration of practice**

1. **Why do you practice intermittent fasting? (Can choose more than 1)**

- To lose weight
- To be healthy
- Religious reasons
- Other (Please specify)

1. **How long do you plan to continue practicing intermittent fasting?**

- < year
- > year
- Five years
- Until I reach certain goals
- For the rest of my life
- I don't know

1. **How did you learn about intermittent fasting?**

- Health programs on TV
- Through social networking sites
- Discussed with health professionals
- Books and magazines.
- Through friends and family.
- Other (please specify)

**Fifth: Use of fasting apps**

1. **1 Which app do you use to count down fasting hours?**

- Zero
- Body Fast
- In Fasting
- LIFE Fasting Tracker
- Window
- Vora
- Fastient
- Fast Habit
- Other (please specify)
- No, I do not use any app for fasting:

**22.2 Why don't you use any of the intermittent fasting applications on your phone?**

- No Arabic applications available
- I didn't know there were applications for intermittent fasting.
- I don't need apps to help me fast.
- Other (please specify)
